# Effects of a 5-Day Back Squat Overreaching Protocol on Strength Performance, Perceived Recovery and Wellness Responses: A Pilot Trial

**DOI:** 10.3390/jfmk10020227

**Published:** 2025-06-13

**Authors:** Lee Bell, Alan Ruddock, Jordan Boriel, Tom Maden-Wilkinson, Steve W. Thompson, Kieran J. Wright, Kieran Burke, David Rogerson

**Affiliations:** 1 School of Sport and Physical Activity, Sheffield Hallam University, Sheffield S10 2BP, UKs.w.thompson@shu.ac.uk (S.W.T.); kieran.burke@shu.ac.uk (K.B.); d.rogerson@shu.ac.uk (D.R.); 2 Loughborough Sport, Loughborough University, Loughborough LE11 3TU, UK; k.j.wright@lboro.ac.uk

**Keywords:** overreaching, strength training, tapering, recovery, one-repetition maximum

## Abstract

Background: The aim of this study was to characterise the performance, perceptual, and wellness responses to a barbell back squat overreaching training protocol. Methods: Eight trained male participants (age = 24.6 ± 2.8 years; relative to body mass back squat one repetition maximum (1-RM) = 1.9 ± 0.4; training experience = 7.0 ± 3.2 years) participated in a 5-day squat OR protocol (SqOR), followed by a 14-day taper. SqOR consisted of five sets of barbell back squats using 80% of daily adjusted 1-RM. A 40% velocity loss threshold was used to determine the set end point. For performance, isometric mid-thigh pull (IMTP) peak force (PF), and countermovement jump (CMJ) PF and jump height; for perceptual, perceived recovery scale (PRS); and for wellness, Hooper Wellness Index (HWI), were recorded at baseline, each day of SqOR, and at select intervals during the taper (POST 1 d, 2 d, 7 d, and 14 d). Follow-up back squat 1-RM testing was conducted at POST 7 d and POST 14 d to determine strength-performance changes relative to baseline. Results: Back squat 1-RM increased by 4.8% at POST 7 d and 5.2% at POST 14 d. IMTP PF increased by 10.3% at POST 7 d and 11.4% at POST 14 d relative to the baseline. CMJ PF and jump height decreased during SqOR but returned to baseline by POST 7 d. PRS and HWI worsened during SqOR, with the greatest impairment occurring on day 3 (PRS = −41.5%; HWI = 34.4%), and did not return to baseline until POST 14 d and POST 2 d, respectively. Conclusions: These findings demonstrate that a short-term period of planned OR improves muscular strength performance, but the duration of the taper influences when peak strength improvements are observed.

## 1. Introduction

Athletes routinely undertake periods of demanding resistance exercise training to enhance the physiological adaptations that underpin a meaningful improvement in performance (e.g., muscular strength and rate of force development) [1]. To achieve the desired outcome, training is organised strategically to achieve peak performance qualities at specific time points relative to the competition schedule [1]. The development of athletic performance is typically approached in a periodised manner, with some training phases designed to “drive” physiological adaptation, and others designed to promote recovery through fatigue management [2,3], i.e., achieve the most efficient gains in adaptation with the lowest degree of fatigue [4]. Consequently, highly demanding training is periodically counterbalanced with adequate phases of recovery so that a marked improvement in athletic performance can be achieved [5]. Strength-trained individuals, however, might experience diminishing improvements in muscular strength as training competency increases [6,7]. A greater relative magnitude of training, therefore, might be required to elicit further physiological adaptations and prepare athletes for the physical demands of competition [8,9,10]. Prolonged periods of highly demanding resistance exercise training without enough recovery, though, can lead to maladaptation [11]. This, of course, presents a logistical challenge for coaches who wish to maximise improvements in well-trained strength athletes whilst mitigating the risk of negative outcomes.

The term “overtraining” (OT) describes the imbalance between training demand and recovery that could result in either diminished performance or an improvement above baseline [12,13]. OT can be intentional (e.g., training camps and impact cycles) or unintentional (e.g., through poor programming, via miscalculation of training and recovery, or by training hard during periods of high non-training stress). In strength sports, it is common for short-term OT to be implemented into the training programme through planned periods of overreaching (OR) [9,14]. During this period of training, there is typically an increase in daily or weekly training volume or relative training intensity, generally for a duration of ~5–7 days [9,14,15]. Moreover, consecutive training bouts or multiple training sessions are undertaken to induce a maximal training stimulus through concentrated loading [9,14,16]. Due to the challenging nature of planned OR, it is common for athletes to experience psychophysiological fatigue both during and in the days/weeks following the bout of OR. This side effect is considered to be an expected part of the training process, and coaches do not perceive such an increase in fatigue to be problematic [17]. Indeed, coaches consider a short-term suppression in performance (and concurrent increase in perceived fatigue) during planned OR as both an anticipated part of the training response and a sign that the training demand is sufficient to achieve a meaningful improvement in performance [14]. Consequently, it is normal for planned OR to be followed by a tapering period where training demand is intentionally reduced to facilitate restoration and to aid performance rebound [18].

The term OT has been used colloquially to describe the “overtraining syndrome” (OTS), a multifaceted medical disorder characterised by an accumulation of training and/or non-training stress resulting in long-term (>4 weeks) decrement in performance capacity [13]. The term OT, therefore, is used to describe periods of challenging phases of training where recovery is (often intentionally) blunted, but it can also refer to the outcome of prolonged performance decrement [13,19]. Such broad vocabulary has led to confusion within the literature surrounding the exact definition and diagnosis of OTS, which is perhaps confounded by the use of “overtraining” as both a verb and noun [20,21,22].

Functional overreaching (FOR) refers to an initial decrease in performance, followed by an improvement in performance relative to baseline after a short period of recovery (often referred to as supercompensation) [23,24,25]. Importantly, FOR occurs when an athlete experiences a decrease in training performance, followed by full restoration and enhanced competition performance within two weeks of appropriate recovery [5]. In strength sports, utilising blocks of planned OR to achieve FOR is common to enhance competition performance [14]. Moreover, planned OR has been used to stimulate FOR as part of a phase potentiation effect, whereby the specific physiological qualities achieved in one block of training enhance the adaptations that occur in the corresponding phase [26]. Whilst planned OR is a ubiquitous practice within strength sports (and many coaches perceive it to be an important aspect of the overall training programme) [14], there is minimal evidence that training designed to induce strength or power-related FOR is superior compared to a more progressive manipulation of training load [24]. Evidence from endurance sports is equivocal, with some studies indicating that planned OR might be a viable method of improving performance [27], whilst others have reported that gains in performance are observed following planned OR, but not when FOR occurs (i.e., when performance improvements are observed but without an initial decrease lasting several days) [23,28].

Non-functional overreaching (NFOR) refers to stagnation or plateau in athletic performance, lasting several days to weeks (~2 to 4 weeks), with no improvement in performance relative to baseline [13,29,30]. The risk of NFOR appears to increase when highly demanding training is undertaken for a prolonged period without sufficient recovery and has been previously observed in strength athlete populations undertaking training camps, frequent competitions and/or excessive training [31,32]. It has been suggested that prolonged exposure to excessive resistance exercise training without sufficient recovery might also lead to the OTS, which is generally considered to be a more severe form of NFOR [13]. The risk of developing NFOR (and hypothetically, OTS) increases when training includes excessive or prolonged high-intensity or high-volume resistance exercise, prolonged training monotony (caused by minimal variation in exercise selection or approach) [33,34], and repeated training to muscular failure [31,32]. It is worth noting that the prevalence of OTS in strength sports is low, with minimal evidence in the existing literature that prolonged periods of resistance exercise training lead to long-term performance decrement [31,32]. Competitive strength athletes frequently report symptoms of general fatigue, musculoskeletal pains, and decreased motivation during periods of underperformance [35]. These symptoms appear to manifest regardless of whether performance impairment is short-term (1 week to 1 month) or long-term (1 to 3 months) [35]. This makes it difficult for coaches to rely on symptoms of performance impairment to dictate training decisions (i.e., premature cessation of training resulting in an absence of performance improvement or late cessation of intensified training resulting in NFOR). This is further compounded by the high degree of inter-individual variability in magnitude and duration of symptoms of performance impairment [32,36].

Previous research has indicated the overall risk of developing NFOR is relatively low following resistance exercise OR, even when training protocols have been designed to induce OTS for the purpose of scientific inquiry [31,32]. Studies that have attempted to induce OTS have adopted either a high-intensity [37,38,39,40,41] or a high-volume [42,43] approach, usually using a squat exercise variation (squat machine or barbell back squat). Findings from these studies have been equivocal, reporting impaired performance [37,38,39,40], no change in performance [43], or improved performance relative to baseline [41]. Such ambiguous findings are likely due to a lack of standardised protocol or methodology and diagnostic criteria, as well as inconsistent follow-up testing. For example, in those studies reporting a decrease in performance (with the exception of Margonis et al., [42]), follow-up performance assessments were performed immediately after completion of the training intervention and not after a recovery period. Therefore, without follow-up testing in the days/weeks following completion of the training protocol, it is not possible to accurately determine that NFOR/OTS occurred. Moreover, given that performance is often suppressed for several days before FOR is achieved, it is plausible that individuals in these studies were experiencing a pattern of normal restorative processes and measurement took place before supercompensation occurred.

Given the ambiguous landscape of research and equivocal findings in strength sport OR domain, it is crucial to better understand the physiological response to intensified resistance exercise training. This is of particular importance to high-performance coaches and athletes that regularly integrate phases of OR into their training programmes to achieve performance improvements or want to avoid long-term performance decrement through the early detection of NFOR. Therefore, the purpose of this study was to examine the feasibility and safety of a pilot OR protocol designed to induce FOR/NFOR in a trained population implementing appropriately timed follow-up assessments to accurately determine training outcomes. Given the challenging nature of the training protocol designed for this study, we hypothesised that whilst some participants would improve performance relative to baseline following short-term performance suppression (FOR), others would experience performance stagnation indicative of NFOR. We did not expect OTS to occur due to the short duration of the protocol.

## 2. Materials and Methods

### 2.1. Experimental Approach to the Problem

**Figure 1:**
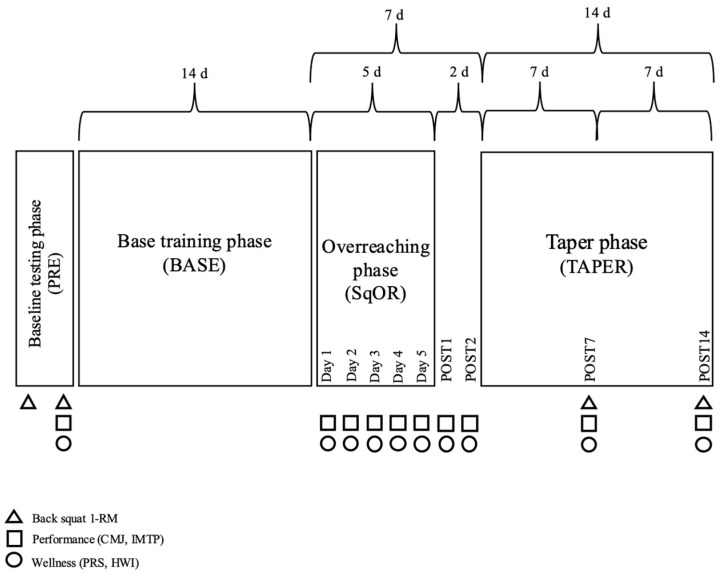
Schematic representation of the study design. CMJ = countermovement jump; IMTP = isometric mid-thigh pull; 1-RM = one repetition maximum; PRS = perceived recovery scale; HWI = Hooper Wellness Index.

A prospective cohort design utilising repeated measures investigated the effects of a pilot protocol on select performance, perceptual, and wellness measures. This study consisted of an initial habituation and baseline testing phase (PRE); a 2-week foundation training phase (BASE); a 5-day “squat overreaching” (SqOR) protocol; and a 2-week taper (TAPER). SqOR consisted of 5 sets of barbell back squats performed each consecutive day, using 80% of daily adjusted 1 RM. All sets were performed until a 40% velocity loss threshold (VLT), facilitating an individualised prescription of training volume in comparison to planned training using a predetermined one-repetition maximum [44]. Previous research has demonstrated that a 40% VLT results in the participant training at (or very close to) concentric muscle failure [45,46,47]. Performance (muscular strength and peak force (PF)), perceptual (recovery status), and wellness measures were recorded at select time points during each phase of the programme (Figure 1).

To assess the safety, feasibility, and appropriateness of the training protocol, a pilot study approach was utilised [48,49]. In physical activity pilot research, small sample sizes may be necessary where there is a limited pool of potential participants, when the research is exploratory in nature, or where data collection might be difficult due to the time required to obtain data [50]. Where a pilot approach has been utilised in OR/OT research [51,52,53] or in exploratory studies investigating the effects of short-term OR on strength performance [16,54,55,56], sample sizes of 6 to 10 participants are common. Considering the nature of the research question in this study, as well as its experimental design, specific inclusion criteria, complexity and novelty of the training protocol, and potential for adverse effects, a sample size of 8 participants was deemed sufficient.

### 2.2. Participants

After institutional ethical approval was granted (ER48910004), eight male participants (mean ± SD; age = 24.6 ± 2.8 years, stature = 175 ± 4 cm, body mass = 83.6 ± 9.9 kg) provided informed consent to participate in the study. Participants had 7.0 ± 3.2 years of resistance exercise training experience and a relative (to body mass) parallel barbell back squat of 1.9 ± 0.4 kg/body mass. Participants were not permitted to undertake any other form of resistance exercise training during the duration of the study period; therefore, any competitive athlete currently within a competition phase was excluded from recruitment. Those who reported a contraindication to exercise (e.g., heart disease and severe musculoskeletal injury) or indicated previous anabolic steroid use were also excluded from the study. Inclusion criteria followed the recommended prerequisites for studies exploring markers of NFOR/OTS [13]. Similar criteria have been used elsewhere [38,46,57,58]. Although participants were well-trained, none had previous experience of undertaking intentional periods of OR.

Stature (cm) was collected at the initial visit, using a commercial height measure system (Seca Leicester, Birmingham, UK). Body mass (kg) was recorded (prior to any physical activity) using a Hawkin Dynamics force plate (Hawkin Dynamics Generation 3; Westbrook, ME, USA). All participants completed a health screening and training-history questionnaire, and informed consent was given before data were collected.

### 2.3. Procedures

#### 2.3.1. Training Programme

The training protocol for this study was organised into three distinct phases (Figure 1). In phase one, participants completed a 14-day base foundation phase (BASE) consisting of two full-body resistance exercise training sessions per week, each separated by ≥72 h (Table 1). BASE was designed by two strength and conditioning coaches (KW, KB) in conjunction with the lead investigator and was designed to ensure participants started SqOR in a similar state of trainability and readiness. The aim of the BASE training phase was not to stimulate additional adaptations or to improve performance per se, but to dissipate fatigue whilst minimising the effects of detraining. This approach has been utilised elsewhere when investigating resistance exercise OR [59].

**Figure 2:**
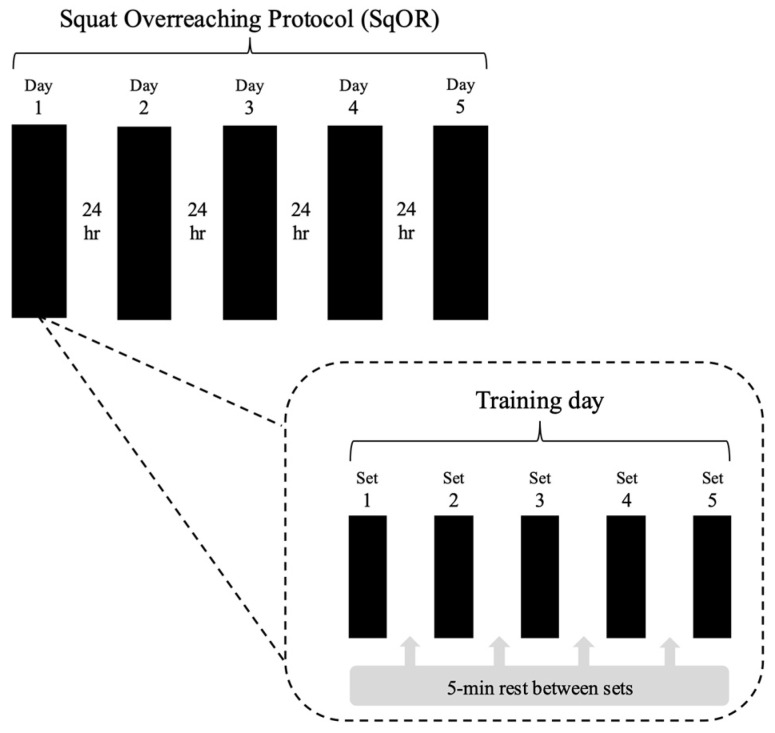
Schematic representation of the SqOR training phase and individual training days.

Phase two of the programme was a 5-consecutive-day SqOR protocol consisting of 5 sets of barbell back squats performed using 80% of daily 1-RM (see Figure 2 for a schematic representation of each training day). Daily load lifted was determined using an autoregulatory approach where participants completed submaximal sets with maximal intent to match the mean concentric velocity (MCV) with baseline values. Training sessions were at the same time of day (±1 h). During the SqOR phase, no other resistance exercise (including upper body training) was permitted. For all sets, participants were instructed to perform as many repetitions as possible, with sets terminated only when a velocity loss of 40% (VL40) was achieved, or when participants reached momentary muscular failure (i.e., despite attempting to, the individual could not complete the concentric portion of the repetition without deviating from the correct form) [60]. A 5 min rest period was provided between sets to standardise inter-set recovery and to reflect the typical rest time recommended for strength training when higher volume loads are utilised [61]. Moreover, longer rest periods were considered more likely to facilitate greater maintenance of barbell velocity [62], allowing for completion of more repetitions in the following set.

When developing complex interventions, there is often a trade-off between answering novel, broad research questions and those that are more narrow or specific [63]. Notably, the design of novel interventions should be adapted to the context and approached in phases, focusing on the feasibility of more critical aspects of intervention in the early stages of trials [63]. Once the primary aspects of the intervention have been assessed, supporting aspects can be revised. Given the challenging nature of the SqOR protocol, a lack of standardised or accepted warm-up protocol for OR research, and the importance of both the physical and psychological aspects of the warm-up (e.g., mental preparation strategies) on readiness to train [64], participants were permitted to complete their preferred warm-up activities rather than a standardised series of exercises. However, participants were asked to self-standardise their warm-up by performing the same routine during each visit. A similar approach to the warm-up has been used elsewhere in the strength training research [65].

All sessions were overseen by the principal investigator and a team of experienced strength and conditioning coaches (JB, KW, and KB), all of whom have previous experience coaching high-performance athletes. Following completion of SqOR, participants were instructed to refrain from all exercise for the following 2 d before commencing the third phase of the programme.

In phase three, participants completed a 14-day taper (TAPER) consisting of two individual full-body sessions each week, separated by ≥72 h (Table 2). Like BASE, TAPER was designed by two strength and conditioning coaches (KW and KB) and the principal investigator, using recommendations provided by Travis and colleagues [66]. TAPER followed a step taper approach where training volume decreased each week over the 14-day period. The aim of the taper was to mitigate fatigue, minimise detraining effects, and enhance recovery before follow-up performance testing. Importantly, the taper should also provide a psychological break from monotonous training [9,67]. Therefore, due to the specialised nature of SqOR (i.e., squat only), and the perceived high risk of participant drop-out (due to the combined effects of training monotony and the duration that each participant had refrained from their normal training by the beginning of the tapering period), the research team decided that TAPER should follow a whole-body-training approach and not just focus on the barbell back squat. That way, participants were permitted to resume a more varied training programme, but with predetermined exercise selection performed at a standardised intensity of effort.

#### 2.3.2. Barbell Back Squat

Participants were permitted to wear a weightlifting belt, knee sleeves, and their preferred footwear, and they could adopt their habitual back squat stance and technique (i.e., high bar or low bar), but they were required to maintain those preferences for the duration of the study [68]. All participants used a standard Olympic weightlifting bar (20 kg Eleiko bar, Eleiko, AB, Halmstad, Sweden). Participants performed the eccentric phase of the back squat under control (~2 s) until a parallel position was achieved, and were instructed to complete the concentric phase of the exercise “as fast as possible, with maximal intent”. Parallel depth was defined as the inguinal fold being level with the musculature of the knee [68]. Participants were given strong verbal encouragement, supervision, and feedback throughout each set to ensure safe and appropriate lifting technique (i.e., proper depth and maximal intent for all repetitions were achieved).

#### 2.3.3. One Repetition Maximum Testing and Load–Velocity Profile

Two individualised load–velocity profiles (LVPs) were conducted at PRE (Figure 1), separated by ≥72 h. The first baseline LVP was conducted to determine the participant’s back squat 1-RM, and the second baseline LVP was conducted to ascertain the MCV at specific percentages of the 1-RM. Procedures followed those outlined by Thompson and colleagues [69]. A follow-up LVP was conducted during TAPER 7 days (POST7) and 14 days (POST14) following completion of SqOR to assess changes in strength performance. Therefore, 1-RM assessment (as part of the LVP) was conducted a total of four times during this study.

Following their individualised warm-up, participants completed five repetitions of the back squat at body mass only (using a wooden dowel); three repetitions at 30%, 40%, and 50% 1-RM; two repetitions at 60%, 70%, and 80% 1-RM; and one attempt at 90% and 100% of the 1-RM. A maximum of five attempts were given to find a true 1-RM. Five minutes of rest were provided between attempts. Participants were instructed to perform the eccentric phase of each attempt with control (~2 s) and the concentric phase of every repetition with “maximal intent and velocity”. MCV from the fastest repetition of each load was recorded. LVP data were collected with a linear position transducer (GymAware RS PowerTool; Kinetic Performance Technologies, Canberra, Australia). The validity, reliability, reproducibility, and sensitivity of this system have been reported elsewhere [70,71], with barbell velocity considered both reliable and valid using this device (ICC > 95%; CV < 5%).

#### 2.3.4. Autoregulation of Training

To ensure an appropriate daily training load for each day of SqOR (i.e., to accommodate for acute changes in readiness), the MCV from the 80% 1-RM (ascertained from the baseline LVP) was used to determine the daily load lifted. To determine load lifted for each day, participants completed 5 reps of the baseline 50% 1-RM, 2 repetitions at 70% 1-RM, and 2 repetitions of 80% 1-RM. The daily load lifted was subsequently adapted if the fastest of the two repetitions at 80% 1-RM was ±0.03 m·s^−1^ from the velocity obtained during the baseline LVP [69].

#### 2.3.5. Velocity Loss Threshold

One of the common features of planned OR is high-volume, high-intensity resistance training performed with a very high level of effort [31,32]. A VL40 was selected for this study, as previous research has shown that performing repetitions with this degree of velocity loss results in the participant training at, or very close to, concentric muscle failure [45,46,47]. The previous literature has elucidated that training with higher velocity loss thresholds leads to larger total training volumes per set and increased mechanical, metabolic, and perceptual disturbance [45]. Higher velocity loss thresholds (such as VL40) lead to reduced peak and mean power across a set of barbell back squats (compared to lower velocity loss thresholds such as ≤20%), likely because of the effects of neuromuscular fatigue as the set progresses nearer to muscular failure [72]. Moreover, VL40 leads to increased muscle damage and impaired mechanical performance compared to lower velocity loss thresholds [73]. For example, Cornejo-Daza [74] reported that VL40 during back squat exercise impaired both jump and squat performance for 24 h post-exercise. Consequently, frequent high velocity loss threshold utilisation might impair both maximal strength and impulse adaptations and increase the risk of NFOR, particularly if insufficient rest is provided between training bouts [74].

The velocity of all repetitions during SqOR was displayed in real time. VL40 was set relative to the fastest repetition of each set (which acted as the reference repetition) [75]. For example, if the fastest repetition for a given set was 0.77 m·s^−1^, the VL40 would be 0.46 m·s^−1^. For all sets, participants were instructed to complete as many repetitions as possible until they could no longer complete the concentric portion of the repetition without deviating from the correct form (momentary muscular failure) or until the GymAware device indicated that VL40 had been reached. Current research (albeit scarce) has observed that during a set of barbell back squats using 80% 1-RM, a total of 5.3 ± 1.5 repetitions are performed by well-trained males to VL40 relative to the fastest repetition of the set [76]. The research team actively provided feedback on velocity and general technique during each repetition and set, and they encouraged each participant to complete all repetitions with maximal intent.

#### 2.3.6. Countermovement Jump

The countermovement jump (CMJ) was performed using Hawkin Dynamics force plates set at a sampling rate of 1000 Hz. Jump height and peak propulsive force were recorded at PRE, before each day of SqOR, at POST1 and POST2, and at select intervals during TAPER (POST7 and POST14) (Figure 1). The reliability of the device has been reported elsewhere [77,78]. Participants were instructed to stand with feet shoulder-width apart and remain motionless for 2 s so that body weight could be accurately determined. This method has previously been determined to be the gold standard for identifying the start of the unweighting phase of a CMJ by detecting a change in body weight by 5 × SD [79]. Each jump was performed with hands on hips to reduce the effects of arm swing. After a countdown of “3, 2, 1, jump”, participants were encouraged to jump with maximal effort, with a self-selected eccentric phase depth. A duration of 60–120 s rest between trials was provided, and 3 trials were performed in total, with the best attempt recorded for that day.

#### 2.3.7. Isometric Mid-Thigh Pull

The isometric mid-thigh pull (IMTP) was performed using guidelines from Comfort and colleagues [80]. Hawkin Dynamics force plates set at a sampling rate of 1000 Hz were used to record peak isometric force at PRE, before each day of SqOR, at POST1 and POST2, and at select intervals during TAPER (POST7 and POST14) (Figure 1). Following an individualised and standardised warm-up, the bar position was adjusted so that it replicated the start of the second pull phase of the clean, resulting in standardised knee and hip angles of 120–135° and 140–150°, respectively. Participants first performed a 50% maximal effort warm-up IMTP for ~3 s. After a brief rest, a second attempt was performed with 75% maximal effort, followed by another attempt with 90% maximal effort, both for ~3 s. Participants maintained an upright torso throughout each attempt (maximum 5–10° forward lean). As pretension is undesirable when assessing IMTP performance, participants were asked to adopt a relaxed position before the start of each test (1 s quiet standing; <50 N change in force). Following a countdown of “3, 2, 1, push”, participants were instructed to “push your feet into the ground as hard and as fast as possible” for <5 s per trial. For all trials (including warm-up attempts), lifting straps were used to ensure that grip strength was not a limiting factor. Each participant completed 3 trials at each testing session, with a 60–120 s rest between attempts. The best attempt of the three trials was used each day.

#### 2.3.8. Perceived Recovery Scale

The perceived recovery scale (PRS) is designed to indicate an individual’s day-to-day level of perceived recovery using a 0–10 scale [81]. Participants were asked to rate their perceived recovery using the following verbal descriptors: “very poorly recovered/extremely tired” was scored 0, “adequately recovered” scored 5, and “very well recovered/highly energetic” was given a score of 10. A score of 0–2 is associated with a decline in performance, 4–6 typically results in similar performance in the corresponding bout of training, and a score of 8–10 represents an expected increase in performance. PRS was recorded upon arrival at the testing laboratory at PRE, before each day of SqOR, at POST1 and POST2, and at select intervals during TAPER (POST7 and POST14) (Figure 1).

#### 2.3.9. Hooper Wellbeing Index

A modified version of the Hooper Wellbeing Index (HWI) [82] was used and consisted of four dimensions: stress, sleep quality, fatigue, and muscle soreness. Each of the four dimensions was scored by participants, with 1 being “very, very good”, and 10 being “very, very bad”. The sum of the four scores was used to calculate the global HWI score. Lower scores indicate better wellbeing, and higher scores indicate poor relative wellbeing. HWI was recorded upon arrival at the testing laboratory at PRE, before each day of SqOR, at POST1 and POST2, and at select intervals during TAPER (POST7 and POST14) (Figure 1).

For both PRS and HWI, participants were provided with a printed version of each scale and asked to point to and verbally state their scores so that the researchers could record them.

### 2.4. Statistical Analysis

As this study was a pilot trial utilising a convenience sampling approach, no sample size calculations were performed [83]. Pilot studies typically have low sample sizes and are often underpowered. Moreover, large variations between participant outcomes can occur, leading to an increased risk of type I statistical errors; therefore, it is not recommended that formal significance testing is undertaken [84]. Current good practice guidelines in pilot research advocate for the use of descriptive statistics and estimation (i.e., confidence intervals (CIs)) to assist with the assessment of precision [85,86,87]. Therefore, all performance and perceptual measures are reported as mean ± SD, as well as CIs and relative percentage change (where appropriate). To indicate the degree of inter- and intra-individual variability for performance and perceptual parameters, the coefficient of variation (CV%) was calculated (CV% = (SD/mean)*100). Smallest worthwhile change (SWC) was used to define the smallest change in practical importance for 1-RM [88,89] and was calculated by multiplying the between-participant SD by 0.2, which is appropriate for highly trained sports participants [88]. Analyses were conducted using SPSS software version 26.0 (SPSS Inc., Chicago, IL, USA).

## 3. Results

**Figure 3:**
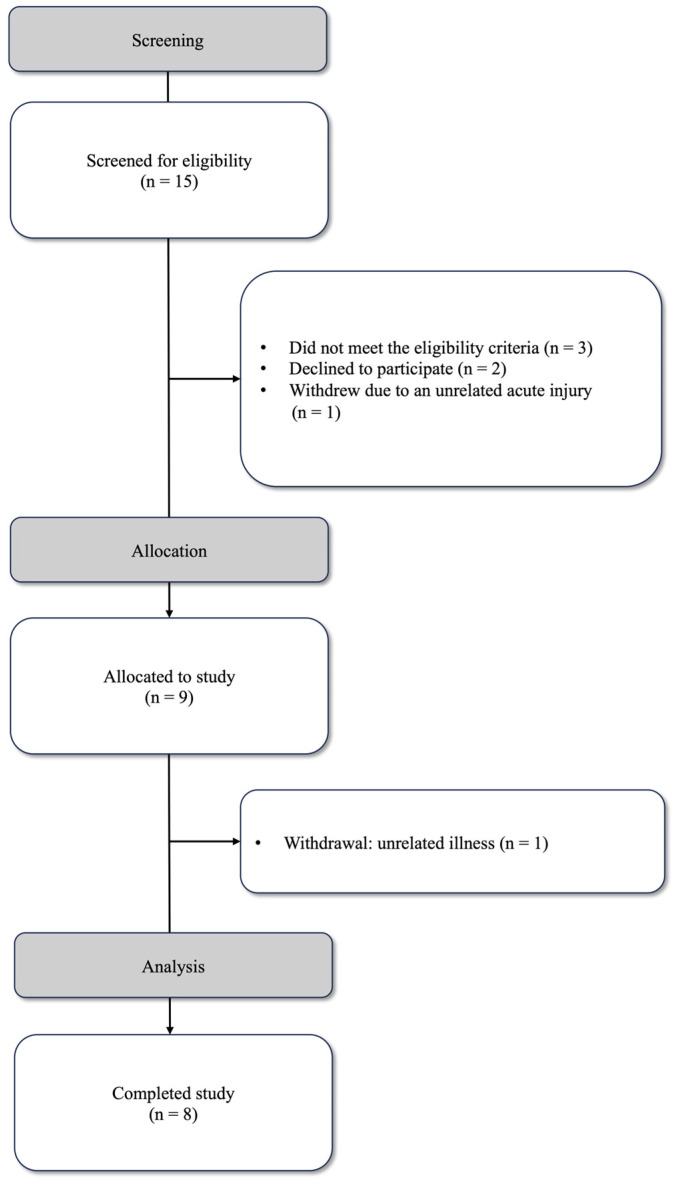
Pilot-study flow diagram.

Fifteen participants were screened for eligibility. Of those, three did not meet the eligibility criteria, as they were elite-standard athletes currently within a competition phase. Two individuals failed to attend initial screening, and one individual withdrew before completing baseline testing due to an injury unrelated to the study. A total of nine participants were recruited for the study; however, one participant withdrew after completing the first day of SqOR because of an unrelated acute illness. The data from the eight participants who completed the study were included in the final analysis (Figure 3).

Compliance for all aspects of the study (defined as total number of training and testing sessions attended for the eight participants) was 100% (each participant attended 11 separate visits over a 6-week period). No participant reported any serious adverse events (e.g., musculoskeletal injury, cardiovascular event, and rhabdomyolysis) due to participation in the study. Participant characteristics are presented in Table 3.

### 3.1. Training Characteristics

**Figure 4:**
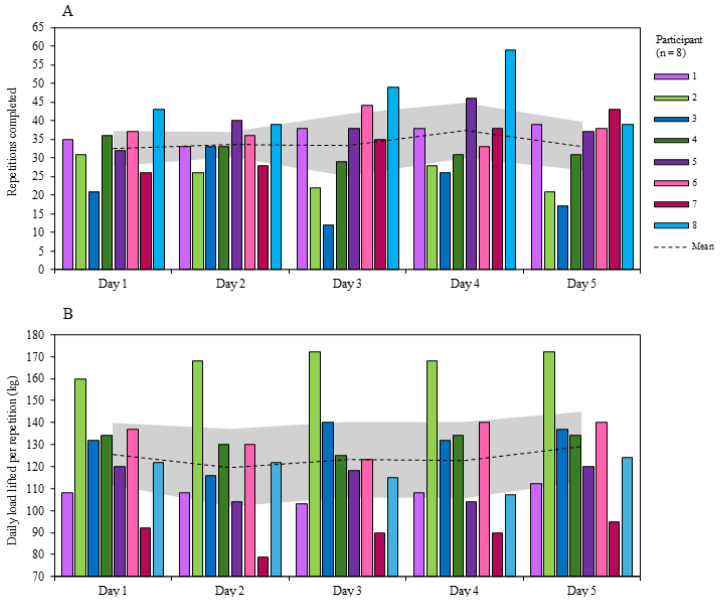
Group mean and individual data for each day of SqOR for (**A**) repetitions completed and (**B**) daily load lifted per repetition (kg). Shaded area indicates 95% confidence intervals.

The mean number of total repetitions completed over the five days of SqOR was 170.0 ± 38.0 [CI = 143.7 to 196.3]. The greatest number of repetitions completed was on day 4 (37.4 ± 10.8 [CI = 29.9 to 44.9]), and the lowest was on day 1 (32.6 ± 6.8 [CI = 27.9 to 37.4]) (Figure 4). The within- and between-participant variability for repetitions completed across the five days was 6.9 to 37.2 and 14.5 to 36.0 CV% respectively.

The mean load per repetition (calculated using 80% of predicted daily 1-RM) for day 1 was 125.6 ± 20.4 kg [CI = 111.5 to 139.7]. The lowest mean load per repetition occurred on day 2 (119.6 ± 25.6 kg [CI = 101.9 to 137.4]), and the greatest on day 5 (129.3 ± 22.7 [CI = 113.5 to 145.0) (Figure 4). Within-participant variability for mean load per repetition across the 5 days was 2.9 to 7.5 CV%, and between-individual variability was 16.2 to 21.4 CV%.

**Figure 5:**
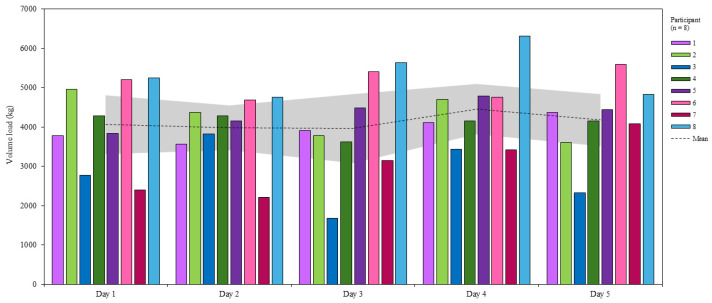
Group mean and individual data for volume load. Shaded area indicates 95% confidence intervals.

Participants completed a total volume load (load × repetitions × sets) of 20,640.4 ± 4441.5 [CI = 17,563 to 23,718] kg over the 5 days of SqOR (Figure 5). The greatest daily mean volume load was on day 4 (4459 ± 929 [CI = 3815 to 5103] kg), and the lowest was on day 3 (3961 ± 1264 [CI = 3084 to 4837] kg). Within-participant CV% ranged from 6.7 to 30.5, and between-participant CV% ranged from 20.6 to 31.0.

### 3.2. Performance Changes

#### 3.2.1. One-Repetition Maximum

**Figure 6:**
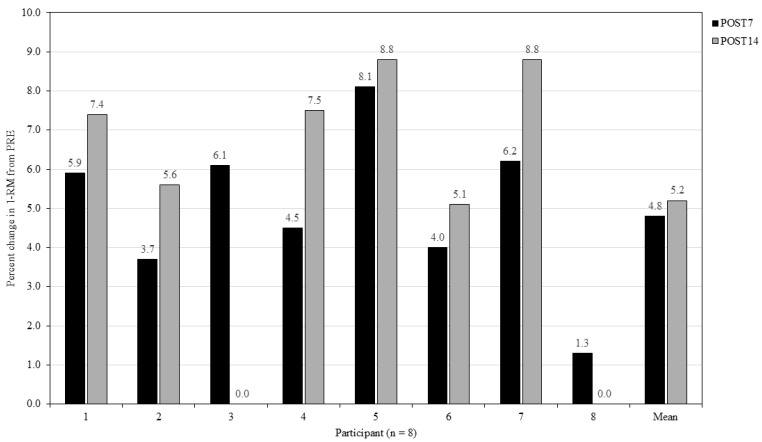
Individual percentage changes in 1-RM relative to PRE at POST7 and POST14. A value of 0.0% represents a return to the baseline 1-RM.

The group mean 1-RM at PRE was 158.9 ± 30.1 [CI = 138.1 to 168.2] kg. At POST7, mean 1-RM was 166.6 ± 30.4 [CI = 145.6 to 176.0] kg, which represented a mean percentage increase of 4.8% (7.7 ± 2.9 kg). At POST14, 1-RM was 167.3 ± 31.0 [CI = 145.8 to 176.8] kg, a mean increase of 5.2% (8.3 ± 5.3 kg) relative to PRE. An increase of 0.4% was observed for 1-RM between POST7 and POST14 (0.6 ± 4.8 kg). At POST7, all participants increased 1-RM relative to PRE (range = 2.0 to 12.0 kg; 1.3 to 8.1%). At POST14, six participants increased 1-RM relative to POST7 (range = 1 to 5 kg; 0.6 to 2.9%). However, 1-RM for two participants returned to PRE (range = −2 to −10 kg; −1.3 to −5.7%). Within-participant CV% ranged from 0.8 to 4.6, and between-participant CV% ranged from 18.2 to 18.9. Figure 6 represents the individual percentage change in 1-RM at POST7 and POST14 relative to PRE. The SWC based on between-participant SD was 6.0 kg (3.8%). At POST7, seven participants exceeded the SWC (range = 7.0 to 12.0 kg), with six participants exceeding SWC at POST14 (range = 9.0 to 13.0 kg). No participants reported improvements in 1-RM that exceeded the SWC between POST7 to POST14 (range = −0.2 to 5.0 kg). One participant, however, did report a decrease in 1-RM performance (−10.0 kg) between POST7 and POST14. MCV for back squat 1-RM at PRE was 0.31 ± 0.06 m·s^−1^. At POST7, velocity for the new 1-RM was 0.28 ± 0.07 m·s^−1^, and at POST14, it was 0.26 ± 0.06 m·s^−1^.

#### 3.2.2. Isometric Mid-Thigh Pull

**Figure 7:**
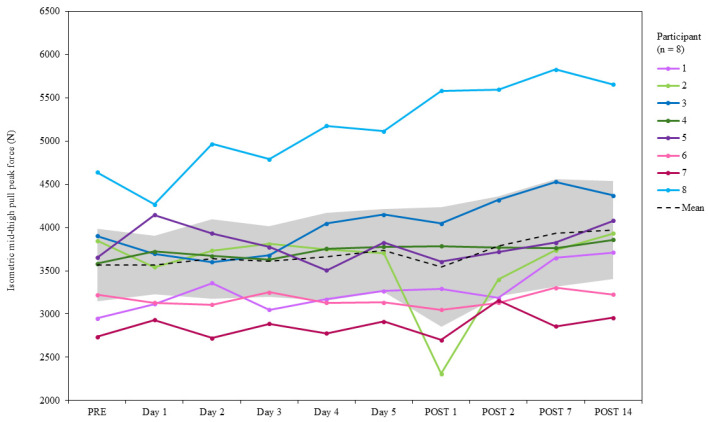
Group mean and individual changes in isometric mid-thigh pull peak force during PRE, SqOR, and TAPER. Shaded area indicates 95% confidence intervals.

The mean IMTP PF at PRE was 3567 ± 602 [CI = 3151 to 3984] N. At POST7, PF increased by 10.3% (3936 ± 899 [CI = 3314 to 4559] N) and by 11.4% at POST14 (3973 ± 817 [CI = 3407 to 4539] N) relative to PRE (Figure 7). The greatest group mean PF was observed at POST14, and the lowest was at POST1 (3547 ± 1000 [CI = 2853 to 4240] N, −0.6% relative to PRE). At POST7, seven participants increased PF relative to PRE (range = 2.5 to 25.6%), and one participant reported a reduction in PF (−2.9%). At POST14, all participants achieved an increase in PF (0.1% to 21.8%) relative to PRE. The within-participant CV% was 2.2 to 13.1, and between-participant variability was 13.7 to 28.2 CV%.

#### 3.2.3. Countermovement Jump

**Figure 8:**
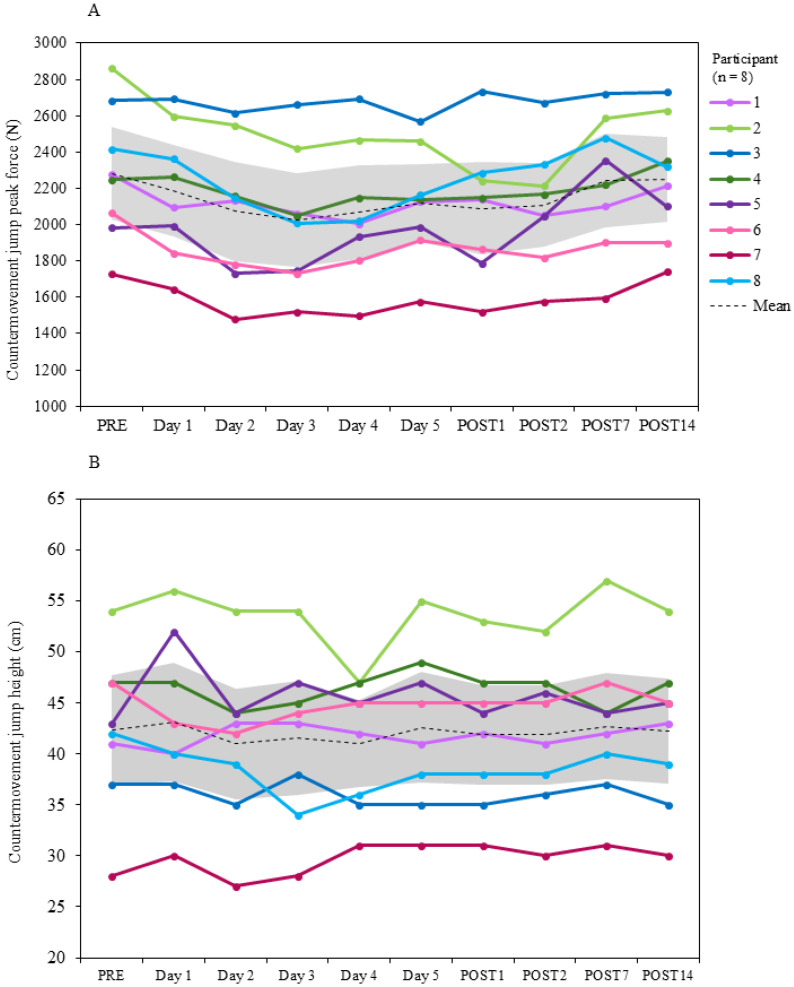
Group mean and individual changes in countermovement jump: (**A**) peak force and (**B**) jump height during PRE, SqOR, and TAPER. Shaded area indicates 95% confidence intervals.

Group mean CMJ PF at PRE was 2283 ± 370 [CI = 2026 to 2539] N, which was the greatest recorded across the study duration. At POST7, PF had decreased to 2244 ± 373 [CI = 1986 to 2502] N, −1.7%; and at POST14, PF was still lower than PRE (2247 ± 337 [2014 to 2481] N; −1.6%). The lowest group mean occurred on day 3 (2024 ± 375 [CI = 1764 to 2284] N; −10.9%) (Figure 8). At POST7, three participants had increased PF relative to PRE (0.4 to 18.8%), and five participants reported a decrease (−1.3 to −9.6%). At POST14, three participants increased PF relative to PRE (0.6 to 8.5%), and five participants had decreased (−2.7 to −8.1%). Within-participant variation was 1.9 to 9.5 CV%, and between-participant variability was 14.7 to 19.0 CV%.

The group mean CMJ height at PRE was 42.4 ± 7.7 [CI = 37 to 48] cm. At POST7, jump height was 42.8 ± 7.6 [CI = 37 to 48] cm, 0.9% relative to PRE; and at POST14, it was 42.3 ± 7.5 [CI = 37 to 47] cm, 0.0%. The lowest recorded CMJ height was on day 2 (41.0 ± 7.8 [CI = 36 to 48] cm; −3.2%) and day 4 (41.0 ± 6.2 [CI = 37 to 45] cm; −3.2%). The greatest jump height occurred on day 1 (43.1 ± 8.4 [CI = 37 to 49] cm; 1.8%) and POST7 (Figure 8). At POST7, four participants increased CMJ height relative to PRE (2.3 to 10.7%), two had returned to baseline (0.0%), and two participants experienced a reduction in jump height (−4.8 to −6.4%). At POST14, three participants achieved an increase in CMJ height (4.7 to 7.1%), two participants returned to baseline (0.0%), and three reported a reduction in jump height (−0.3% to −7.1%) relative to PRE. Within-participant variation was 2.5 to 5.8 CV%, and between-participant variability was 15.0 to 19.4 CV%.

### 3.3. Perceived Recovery and Wellness

#### 3.3.1. Perceived Recovery Scale

**Figure 9:**
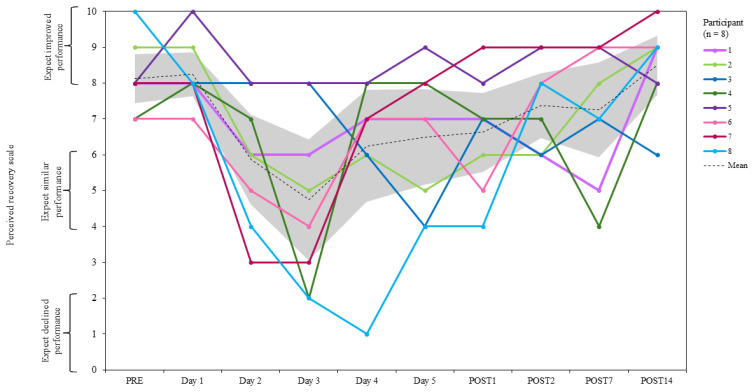
Group mean and individual changes in perceived recovery scale during PRE, SqOR, and TAPER. Shaded area indicates 95% confidence intervals.

The mean PRS score at PRE was 8.1 ± 1.0 [CI = 7.4 to 8.8]. At POST7, PRS decreased to 7.3 ± 1.9 [CI = 5.9 to 8.6], −10.8%; and at POST14, it increased to 8.5 ± 1.2 [CI = 7.7 to 9.3], 4.6% relative to PRE. The lowest recorded mean PRS score was on day 3 (4.8 ± 2.4 [CI = 3.1 to 6.4]; −41.5%), and the greatest was on POST14 (Figure 9). Within- and between-participant variability were from 8.3 to 54.3 and from 10.7 to 51.3 CV%, respectively.

#### 3.3.2. Hooper Wellbeing Index

The group mean score for global HWI (the summation score for all dimensions) at PRE was 11.5 ± 2.8 [CI = 9.5 to 13.5]. Global HWI was lower than PRE at both POST7 (10.4 ± 4.2 [CI = 7.5 to 13.3]; −9.8%) and POST14 (9.9 ± 3.4 [CI = 7.5 to 12.2]; −14.1%). The greatest global HWI score occurred on day 3 (15.6 ± 4.1 [CI = 12.8 to 18.4]; 34.4%), and the lowest score occurred on POST14. Within-participant variability was 17.1 to 40.1 CV%, and between-participant variability was 17.3 to 40.2 CV%.

The group mean score for HWIsleep at PRE was 3.4 ± 1.1 [CI = 2.6 to 4.1]. HWIsleep was lower than PRE at both POST7 (2.9 ± 1.5 [CI = 1.9 to 3.9]; −14.8%) and POST14 (2.9 ± 1.6 [CI = 1.2 to 4.5]; −14.8%). The greatest HWIsleep score occurred on POST1 (4.0 ± 1.6 [CI = 2.9 to 5.1]; 18.5%), and the lowest score occurred on POST7 and POST14. Within- and between-participant variability were from 27.2 to 59.2 and from 31.4 to 63.2 CV%, respectively.

The group mean score for HWIstress at PRE was 3.1 ± 1.7 [CI = 1.9 to 4.3]. HWIstress was lower than PRE at POST7 (2.8 ± 1.5 [CI = 1.7 to 3.8]; −12.0%) but returned to PRE levels at POST14 (3.1 ± 1.6 [CI = 1.5 to 4.8]; 0.0%). The greatest HWIstress score occurred on day 1 (3.5 ± 1.9 [CI = 1.6 to 5.4]; 12.0% higher than PRE), and the lowest score occurred on POST2 (2.4 ± 0.9 [CI = 1.5 to 3.3]; −24.0% relative to PRE). Within-participant variability was 13.1 to 63.3 CV%, and between-individual variability was 30.2 to 55.3 CV%.

The group mean score for HWIfatigue at PRE was 2.6 ± 0.7 [CI = 2.1 to 3.1]. HWIfatigue was the same as PRE at POST7 (2.6 ± 1.2 [CI = 1.8 to 3.4]; 0.0%), but it decreased to 2.0 ± 0.8 [CI = 1.2 to 2.8; −23.8%] at POST14. The greatest HWIfatigue score occurred on day 3 (4.0 ± 1.6 [CI = 2.4 to 5.6], 47.6% increase from PRE; and the lowest score occurred on POST14. Within-participant variability was 16.6 to 60.4 CV%, and between-participant variability was 27.3 to 50.6 CV%.

**Figure 10:**
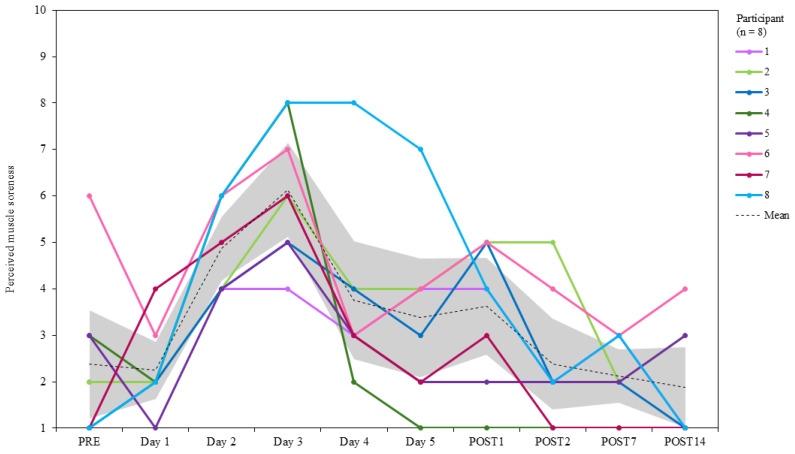
Individual changes in perceived muscle soreness (HWIsoreness) during PRE, SqOR, and TAPER.

The group mean score for HWIsoreness at PRE was 2.4 ± 1.7 [CI = 1.2 to 3.5]. HWIsoreness was lower than PRE at POST7 (2.1 ± 0.8 [CI = 1.5 to 2.7]; −10.5%) and POST14 (1.9 ± 1.2 [CI = 0.6 to 3.1]; −21.1%) (Figure 10). The greatest HWIsoreness score occurred on day 3 (6.1 ± 1.5 [CI = 4.7 to 7.6]; 257.5% increase from PRE), and the lowest score occurred on POST7. Within- and between-participant variability were from 31.9 to 94.6 and from 20.3 to 70.9 CV%, respectively.

## 4. Discussion

The purpose of this study was to explore the feasibility and safety of a 5-day back squat OR protocol (SqOR). This study was the first of its kind because it shows that undertaking consecutive days of high-intensity, high-volume barbell back squat OR using a 40% VLT to dictate set end point results in practically meaningful strength improvements. It also shows that improvements in strength follow an individualised peaking profile, which has important contextual implications for how the post-OR taper might be implemented into the training programme. To ensure our methods were robust, we used a highly standardised programme of training, consisting of a foundation training phase, a 5-day OR phase, and a tapering phase. We incorporated an autoregulated loading approach to accommodate for daily changes in readiness, and we integrated performance and perceptual assessments at regular timepoints to monitor acute alterations in training status.

In line with good practice for pilot research [48,49], this study aimed to evaluate elements of SqOR before future fully powered randomised controlled trials can take place. We hypothesised that the physiological response to SqOR would be variable, with some participants achieving a meaningful improvement in strength performance relative to baseline (i.e., FOR) and others experiencing a decrease in performance lasting several days or weeks, with no observed improvements (i.e., NFOR). We did not expect OTS to occur given the duration of the protocol. The main findings of this investigation are that (1) completion of SqOR followed by a taper resulted in improved muscular strength that surpassed the SWC, (2) muscular-strength gains followed an individualised peaking profile, and (3) undertaking consecutive bouts of resistance exercise training whilst under-recovered did not result in performance decrement or any other adverse effect. A plausible explanation for the improvement in muscular strength observed in this study is that the protocol undertaken was sufficient to induce highly specific neuromuscular adaptations in a trained population but not excessive enough to cause maladaptation.

In line with similar research [37,38,39,40,41,43,57], the training-specific criterion measure used to determine performance change in this study was back squat 1-RM. At POST7, strength improvements were observed for all participants relative to baseline. At POST14, six participants observed an additional, albeit smaller, 1-RM increase, whilst strength gains had returned to baseline for two participants.

In well-trained populations (such as those recruited for this study), even the smallest of performance improvements are of primary importance [90]. It is generally accepted that highly trained individuals improve their strength performance at a lower magnitude compared to untrained individuals [6,91]. Therefore, the SWC provides important information relating to real-world performance changes. This approach has been used in other studies aiming to detect meaningful changes in strength performance following a period of planned OR and is considered an effective way to quantify performance changes, particularly during intense periods of training [92,93]. In this study, we estimated the SWC (calculated from baseline 1-RM testing) to be 6 kg. At POST7, seven of the eight participants achieved improved 1-RM performance that surpassed the SWC, with six participants maintaining improvements that surpassed the SWC at POST14. Therefore, completion of SqOR followed by a period of tapering augmented strength improvements that were practically meaningful. Importantly, the additional improvement in 1-RM experienced by some participants between POST7 and POST14 did not surpass the SWC, suggesting that whilst a longer taper might have been beneficial for some participants to reach “peak” improvements in strength (based on absolute change in load lifted), the magnitude of change was not practically meaningful. One participant did experience a reduction in 1-RM between POST7 and POST14 that surpassed the SWC (−10.0 kg), suggesting that a meaningful level of detraining had occurred.

FOR occurs when an athlete experiences a temporary (~days) decrease in performance, followed by enhanced performance within 1–2 weeks [5,16,23]. There is currently no accepted threshold by which performance improvements are constituted as FOR [13]. To be more precise, *any* improvement in the criterion measure of performance could be considered FOR based on current consensus, as long as it is preceded by a short-term period of performance decrement [13,94]. This is likely due to the multifactorial nature of supercompensation and a lack of accepted gold-standard test or assessment to differentiate FOR from NFOR [32]. Nevertheless, in an applied strength and conditioning environment, coaches seek training-related changes that have practical relevance, e.g., whether the change in any given parameter is greater than the smallest practical or meaningful change [95].

In this study, improvements in back squat 1-RM were observed only after an initial decrease in training readiness (as indicated through a reduction in daily load lifted, CMJ height, and PF; and perturbations in select measures of perceived recovery and wellness). Moreover, all participants observed a temporary reduction in daily load lifted at varied time points during SqOR. Whilst there is evidence of a supercompensatory effect in maximal strength (as evidenced by 1-RM improvements relative to baseline), there was no indication that FOR had taken place based on current definitions. Indeed, for FOR to have occurred, a reduction in 1-RM would be expected at POST7 that may have been sustained until POST14. However, all participants had improved muscular strength above baseline at that point. Therefore, it is more likely that the transitory and temporary perturbations in daily readiness (i.e., daily load lifted and CMJ) were reflective of the acute fatigue generally observed during the adaptive response following high-effort resistance exercise training [96,97]. As mentioned, NFOR occurs when athletes undertake a period of intensified training, resulting in stagnation or plateau in athletic performance lasting several days to weeks (~2 weeks), with no improvement in performance relative to baseline [13,29,30]. As all participants in this study reported an increase in back squat 1-RM relative to baseline, no cases of NFOR occurred.

Undertaking regular high-volume and high-intensity effort training can lead to performance improvements above baseline but also increases susceptibility to NFOR [32]. The risk of NFOR developing further increases when repetitive efforts close to muscular failure are performed [31,98], where exercise-induced muscle damage occurs [99,100], where there is low variation in exercise selection [32], or where there is insufficient recovery between bouts of training [101]. Indeed, several previous studies have reported performance decrements where a high-volume training protocol was performed with repeated high effort using only a single resistance exercise [37,39,40,43,57]. It must be noted though that in those studies reporting a decrease in performance, follow-up testing was performed immediately after completion of the training intervention and not following a recovery period or taper. The absence of NFOR observed in our study is likely, in part, due to follow-up performance testing taking place after a planned taper, which is indicative of real-world training practice [2,9]. The taper itself is considered to be an important component of the adaptive process, as training during this phase is organised in a way that mitigates fatigue and facilitates physiological adaption [18,102,103].

In this study, two athletes achieved peak strength performance at POST7 before returning to baseline, whereas six participants did not peak until POST14. Importantly, if 1-RM had only been assessed at POST14, (i.e., no 1-RM testing occurred at POST7), NFOR might have been (albeit erroneously) determined based on a lack of performance improvements following SqOR. Therefore, it is important that coaches and sports scientists consider when testing occurs to best determine performance changes. Indeed, the timing of performance change can vary between athletes despite similar training demands [20], with up to <4 weeks of tapering required by some athletes to experience strength improvements following intentional OR [18,104]. Previous research has elucidated variable adaptation kinetics where the time course of the adaptive process, e.g., the duration required to observe peak performance gains following a period of planned OR, varies between athletes, even when undertaking the same training protocol [105,106]. Therefore, findings from this study might have important implications for how post-OR testing is scheduled and demonstrates that the duration of the taper influences muscular strength outcomes following a period of OR.

In this study, we incorporated several performance and perceptual assessments at select time points throughout the training programme. These assessments were, in part, informed by previous research [13,30,31,32] but also by real world high-performance strength-coaching practice [14,17]. When training to muscular failure, suppression of maximal strength and alteration in force-generating characteristics can require <72 h to resolve [97,107,108]. Where exercise-induced muscle damage has occurred (due to extremely high training volumes, eccentric loading, or unaccustomed exercise), recovery can require ≤96 h [109,110]. Therefore (based on the design of SqOR), we anticipated that participants would be under-recovered during (and after) the training protocol, and that by incorporating daily testing, fatigue, recovery, and readiness to train could be appropriately monitored, both objectively and subjectively [35].

In strength sports and resistance-training programmes, indicators of training readiness often include objective measures, i.e., 1-RM, barbell velocity metrics (e.g., MCV), CMJ height, and CMJ/IMTP force metrics [111,112,113], as well as subjective measures of perceived recovery and wellness [114,115]. The combination of both objective and subjective information permits the coach to make informed decisions regarding how training might be adapted day to day (i.e., autoregulation) [13,92]. Moreover, there are often large correlations between objective and subjective strength performance assessments [114], allowing a degree of flexibility and individualisation when monitoring readiness and recovery. In this study, CMJ PF was lower than baseline each day during SqOR, as well as at specific points during the taper, suggesting that PF derived from CMJ was sensitive enough to detect fatigue and alterations in daily training readiness. Indeed, previous research has indicated that CMJ PF and jump height provide the most consistent measure of neuromuscular fatigue during periods of OR, providing the coach with an accurate and sensitive assessment to monitor performance and avoid maladaptation [42,116,117]. However, whilst consistent reductions in jump performance were observed in this study, NFOR did not occur. Moreover, reductions in jump performance did not always correspond to the daily load lifted or volume load completed during SqOR. Therefore, caution must be taken when using jump performance in isolation to assess training readiness, as premature termination of training based on reductions in jump performance alone might negatively impact athletes achieving performance gains. Further, based on our findings, a reduction in jump force or height during SqOR was the result of acute fatigue rather than an NFOR, as jump performance returned to baseline for most participants within <7 days of completing SqOR.

Whilst decrements in CMJ PF were observed during SqOR, IMTP PF increased most days and improved relative to baseline at both POST7 and POST14. IMTP PF is typically used as an indicator of acute neuromuscular fatigue and preparedness for training due to its potential sensitivity to fatigue [118,119]. However, there is a large variance and lack of agreement between IMTP and dynamic strength changes following resistance exercise-training interventions, likely due to discrete neuromuscular domains [120]. Participant 8 experienced the greatest increase in IMTP PF (25.6% at POST7) relative to baseline but also reported the smallest improvement in back squat 1-RM. Interestingly, participant 8 also recorded the lowest PRS score across the whole study (day 4) and highest perceived muscle soreness (occurring on days 3 and 4 of SqOR). Similar results have been observed elsewhere [121], where no significant alterations in IMTP PF were observed following completion of either a high-volume or high-intensity back squat protocol, even though markers of muscle damage were elevated. Based on findings from our study and previous research, PF might not be sensitive enough to detect meaningful neuromuscular impairment during planned OR and did not appear to relate to daily measures of squat performance (daily load lifted and volume load). Therefore, coaches should be cautious when using IMTP PF to assess daily readiness during periods of intensified training.

In this study, participants reported a large decline in perceived recovery and wellness scores during SqOR, suggesting under-recovery and diminished preparedness between training bouts. Participants reported large increases in perceived muscle soreness and fatigue (both of which peaked on day 3), which appeared to correspond with jump performance (which was lowest on day 3). Stress and sleep were mostly unchanged relative to baseline during SqOR, although stress was higher than baseline on day 1 of SqOR. Participants had a lack of previous experience implementing intentional OR in their training programme, and the increased stress reported before SqOR might have reflected a “pre-microcycle anxiety” or threat state where athletes considered the protocol to be challenging or unachievable [122]. This is, however, speculative, and, to date, there has been no research that has explored perceptions of planned OR from the perspective of the athlete. All subjective measures of recovery and wellness were resolved by the completion of the study, suggesting any negative alterations in perceived recovery and wellness were indicative of a acute fatigue rather than chronic maladaptation [34,107].

The previous literature has indicated that the PRS can detect increased fatigue and muscle damage following periods of high-volume resistance exercise planned OR and therefore might accurately assess the readiness of an athlete during training sessions across an intensive microcycle. Following high-intensity back squat exercise, PRS appears to be more sensitive to fatigue than objective measures such as CMJ jump height and IMTP PF [123]. Subsequently, monitoring perceived stress and recovery might help prevent NFOR by warning coaches of reduced recovery during periods of highly demanding, concentrated loading [124]. Nevertheless, during periods of planned OR, training is organised in a manner that recovery between bouts is purposefully impaired. Therefore, temporary decrement in perceived recovery should be expected given the demands of the training cycle. It is unsurprising that participants of this study reported diminished recovery and wellness given the design of SqOR. However, NFOR did not occur in this study, suggesting that diminished recovery reported by participants was not a warning sign of maladaptation and indicative of the normal adaptive response to highly demanding training. It is also worth noting though that perceived recovery is also highly individualised, with research reporting inconsistencies between daily performance measures (back squat MCV and CMJ height) and perceived recovery [113,125].

### 4.1. Limitations

Identifying and discussing the limitations of a pilot study contextualises the importance of its findings [126]. Moreover, acknowledging limitations in pilot trials can improve the quality of future definitive controlled research studies [85]. Therefore, the following information should be considered during the development phase of future research projects intending to utilise resistance exercise training planned OR protocols.

This study aimed to examine the feasibility and safety of a pilot planned OR protocol in a trained population and was not powered to detect statistical difference. In pilot research, it is not recommended that formal significance testing is undertaken [84]. Consequently, descriptive statistics were used on primary and secondary outcome data in line with recommendations for pilot research [49]. The emphasis on descriptive statistics should not be viewed as a limitation, but rather to assess the feasibility of the protocol. Whilst SqOR was designed to induce a state of OR for the purpose of scientific inquiry, which was (in some regards) observed through the reduction in daily readiness and diminished perceived recovery, no cases of NFOR occurred. Therefore, it can be argued that the protocol in this pilot was not sufficiently challenging to induce long-term maladaptation. Despite this, the positive performance effects observed suggest that consecutive days of high-volume, high-intensity squats could be a viable approach for inducing muscular strength improvements in trained individuals. It must be noted though that whilst specific inclusion criteria were used to determine the training status of participants in this study, the baseline values across the participant sample were dissimilar (e.g., training history and baseline relative strength characteristics).

SWC was used in this study to contextualise the magnitude of performance change; however, it must be acknowledged that the method by which SWC was calculated in this study might have limitations, since the magnitude of the SWC is affected by the homogeneity of the participant population (i.e., the greater the homogeneity, the lower the SWC) [127]. In this study, the range of PRE 1-RMs was relatively broad (158 ± 30.1; range = 113 to 215 kg); therefore, future research exploring the effects of SqOR on 1-RM would benefit from a larger, more homogeneous pool of athletes to reduce the potential for error.

A 40% VLT was implemented into SqOR to ensure each working set was performed close to muscular failure and to standardise (as much as was practically possible) the degree of effort between participants (as well as for the same participants on different training days). We acknowledge though that the number of repetitions completed at a given load within a specific VLT can be variable [128], likely due to individual athletes’ strength–endurance abilities. Moreover, we acknowledge that even the magnitude of velocity loss achieved during a given set cannot necessarily inform proximity to failure during resistance exercise training [129]. Nevertheless, using a VLT of 40% mitigated the risk of injury that could have been caused by repeatedly “bailing” the barbell by training to absolute muscular failure. Moreover, the reduction in neuromuscular function (and concomitant increase in fatigue) experienced following a set to failure is not dependent on the number of repetitions completed per se, but the magnitude of velocity loss (and as such, the degree of effort applied) [4]. Consequently, using a high VLT such as the one in this pilot increased the chances of altered perceptual, metabolic, and mechanical output, which, hypothetically, should have increased the risk of NFOR occurring [128,130].

Finally, whilst it is not a limitation of the study per se, the protocol implemented for this pilot focused solely on the barbell back squat exercise, and no other resistance exercise was permitted during the SqOR phase. Therefore, the effects of such training within a holistic training programme have not been determined, and caution must be taken when applying these results within a practical training environment.

### 4.2. Practical Applications

A growing body of evidence suggests that the prevalence of NFOR is low within strength-trained populations. Further, there is little evidence that true OTS has occurred in athletic populations following an intensive period of resistance exercise training [12,31,32]. Findings from this pilot suggest that a period of planned OR consisting of consecutive days of high-volume, high-intensity back squats induces performance improvements and does not result in NFOR or any other adverse effects. Nevertheless, caution must be taken when attempting to contextualise these findings within a practical training environment, and further research is required to understand the utility of such training in a real-world setting.

It is common for strength athletes to intentionally implement OR within specific phases of the competition training programme; therefore, future research should continue to examine how such training should be organised to achieve an optimal adaptive response. This study, in part, shows that undertaking consecutive days of resistance exercise could be a strategy to induce strength gains within a short period of time. Consequently, future studies must continue to investigate the mechanisms that underpin FOR to better understand the factors that contribute to the adaptive response.

A challenge that future research might have in trying to induce NFOR (for the purpose of scientific inquiry) is the ethical considerations attached to developing more challenging resistance exercise protocols. To examine the maladaptive response to resistance exercise, future research will be required to undertake more challenging protocols, which, of course, might carry inherent and additional risks. Consequently, research ethics committees must be cognisant that protocols such as SqOR are not as likely to result in maladaptation as originally assumed.

## Figures and Tables

**Table 1:** BASE training programme.

Training Session 1	Training Session 2
BB back squat (2 × 8 @ 75% 1-RM)	BB back squat (2 × 8 @ 75% 1-RM)
Romanian deadlift (2 × 5 @ 75% 1-RM)	BB bench press (2 × 8 @ 75% 1-RM)
DB shoulder press (2 × 8 @ 75% 1-RM)	Goblet lateral lunge (2 × 6 @ 75% 1-RM)
Pull-ups (2 × 2 RIR)	Single arm DB row (2 × 8 @ 75% 1-RM)
Calf raises (2 × 8 @ 75% 1-RM)	Pallof press and rotate 2 × 8
Close grip bench press (2 × 8 @ 75% 1-RM)	Banded ankle knee rockers (2 × 2 RIR)
Abdominal curls (2 × 2 RIR)	KB hip openers (2 × 2 RIR)

BB = barbell; DB = dumbbell; KB = kettlebell; RIR = repetition in reserve.

**Table 2:** TAPER training programme.

Training Session 1	Training Session 2
BB back squat (3 × 6 @ 75% 1-RM)	BB back squat (3 × 6 @ 75% 1-RM)
Romanian deadlift (3 × 5 75% 1-RM)	Glute ham raise (3 × 8 @ 75% 1-RM)
DB shoulder press (3 × 8 @ 75% 1-RM)	BB bench press (3 × 6 @ 75% 1-RM)
Straight-arm pull (2 × 8 @ 75% 1-RM)	Single arm DB row (3 × 8 @ 75% 1-RM)
Band pull-apart (3 × 2 RIR)	DB lateral raise (3 × 8 @ 75% 1-RM)
Close grip bench press (3 × 8 @ 75% 1-RM)	BB reverse curls (3 × 8 @ 75% 1-RM)

BB = barbell; DB = dumbbell; RIR = repetition in reserve.

**Table 3:** Baseline participant characteristics.

Characteristic	Mean ± *SD*
Age (y)	24.6 ± 2.8
Stature (cm)	175 ± 4.0
Body mass (kg)	83.6 ± 9.9
Resistance training experience	7.0 ± 3.2
One-repetition maximum:	
Absolute (kg)	158.0 ± 30.1
Relative to body mass	1.9 ± 0.4
Isometric mid-thigh pull peak force (N)	3568 ± 602
Countermovement jump:	
Peak force (N)	2283 ± 370
Height (cm)	42 ± 8.0

## Data Availability

The datasets used and/or analysed during the current study are available from the corresponding author on reasonable request.

## Abbreviations

The following abbreviations are used in this manuscript:

1-RM	One-repetition maximum
CMJ	Countermovement jump
CV	Coefficient of variation
FOR	Functional overreaching
HWI	Hooper Wellness Index
IMTP	Isometric mid-thigh pull
LVP	Load–velocity profile
MCV	Mean concentric velocity
NFOR	Non-functional overreaching
OR	Overreaching
OTS	Overtraining
PF	Peak Force
PRS	Perceived recovery scale
SqOR	Squat overreaching protocol
SWC	Smallest worthwhile change
VLT	Velocity loss threshold
